# Health literacy, health outcomes and community health worker utilization: a cohort study in HIV primary care

**DOI:** 10.1186/s12913-022-08634-7

**Published:** 2022-10-17

**Authors:** Christina E. Freibott, Linda S. Sprague Martinez, Serena Rajabiun, Mari-Lynn Drainoni

**Affiliations:** 1 Department of Health Law, Policy & Management, Boston University School of Public Health, 715 Albany Street, Talbot Building, Boston, MA 02118 USA; 2 Center for Innovation in Social Work and Health, Boston University School of Social Work, Boston, MA USA; 3 Macro Department, Boston University School of Social Work, Boston, MA USA; 4 Department of Public Health, Zuckerberg College of Health Sciences, University of Massachusetts, Lowell, MA USA; 5 Section of Infectious Diseases, Department of Medicine, Boston University School of Medicine, Boston, MA USA; 6 Evans Center for Implementation and Improvement Sciences, Boston University, Boston, MA USA

**Keywords:** People with HIV, Community health worker, Health literacy, Coaching

## Abstract

**Background:**

People with HIV (PWH) have complex needs, and those with limited health literacy consistently have poorer HIV-related knowledge and health outcomes. One strategy to facilitate better outcomes for PWH is the inclusion of community health workers (CHWs) into care teams. This cohort study examines the effect of health literacy on clinical outcomes and utilization of CHW services among PWH enrolled in a CHW intervention. The secondary aim is to characterize most common purposes of CHW encounters.

**Methods:**

PWH (*n* = 209) enrolled in a CHW intervention with completed 6-month follow-up evaluation visits were included. Health literacy level was measured at baseline with the BRIEF tool and categorized into inadequate, marginal, and adequate health literacy. Adjusted logistic regressions assessed the effect of health literacy on viral load suppression, HIV primary care visits at 6-month follow-up, CHW utilization and purpose of CHW encounter. Purpose of CHW encounters included logistical support, accompany to appointment, transportation coordination, concrete services, coaching, and emotional support. Linear regression assessed the association between purpose of CHW encounters and CHW utilization.

**Results:**

Individuals with inadequate health literacy were more likely to receive coaching from CHWs (*p* = 0.029), and individuals with marginal health literacy were more likely to have an HIV primary care visit at 6 months (*p* = 0.044). Individuals receiving transportation coordination, concrete services, coaching, and emotional support had more total CHW encounters.

**Conclusions:**

Purpose of encounter was highly correlated with frequency of CHW encounters, while health literacy status was not. This suggests individuals receiving these services require more assistance from CHWs, regardless of health literacy level. Training CHWs to conduct comprehensive social needs assessment and screening for risk factors at the initial visit with clients can identify resources and guide CHW service delivery as part of the care team.

**Supplementary Information:**

The online version contains supplementary material available at 10.1186/s12913-022-08634-7.

## Background

Health literacy is the ability to understand and use health-related information to inform decisions [1, 2]. Limited health literacy has deleterious health impacts, including difficulty interpreting pharmaceutical labels or adhering to medication regimens, poorer overall health status and higher mortality [3]. For the 80 million adults in the United States with limited health literacy, access to healthcare is impeded, with decreased utilization of preventative services such as mammography screening and influenza immunizations [3].

Health literacy disproportionately impacts people of color and those of low socioeconomic status who are also over represented in the population of people with human immunodeficiency virus (HIV) [1]. Limited health literacy can lead to poor medication adherence, which is especially crucial for people with HIV (PWH) taking antiretroviral therapy (ART) to manage disease progression and achieve viral suppression [4]. One study reported that for PWH who identify as Black, low health literacy was correlated with lower CD4+ T cell counts, indicating a weaker immune system [5]. Furthermore, PWH with limited health literacy may be less comfortable communicating with providers or unwilling to ask questions, which can be detrimental to the quality of care received [6]. PWH with limited health literacy are less likely to receive information from their providers that they can understand, which hinders their ability to manage HIV and prevent transmission [6].

One strategy to facilitate better health outcomes for PWH is the inclusion of community health workers (CHWs) into care teams at HIV clinical care locations. CHWs are public health workers who provide culturally relevant health education, community outreach, care coordination, social support, and advocacy [7]. The role of CHWs in improving health outcomes is well reported for a variety of chronic conditions, such as diabetes, asthma, and hypertension [8–10]. More recently, CHWs have been studied in the HIV primary care setting [11, 12]. The strategy of using CHWs is important to support HIV primary care providers through a variety of tasks, which can include assistance in creating appointments, arranging transportation, and home visits [11, 13, 14]. Several studies have investigated the positive effect that CHWs have on care delivery for PWH, including better linkage to care, retention in care, ART adherence, and viral suppression [15–18]. While the role of CHWs assisting PWH has been described broadly, few studies exist that specify the most frequently utilized CHW services or details of CHW-patient encounters in this population. Additionally, health literacy has been reported to have an effect on patient satisfaction with CHWs, which could impact their willingness to use CHWs during treatment [19]. Further, the impact of health literacy on CHW utilization and health outcomes has yet to be explored.

A recently published evaluation of the current intervention (Drainoni et al.,2020) assessed how increased utilization of CHWs within HIV primary care clinics impacted health outcomes in the first 6 months of engagement with a CHW [11]. Participants had 11 encounters with CHWs on average in the first 6 months. Significant findings included improvements primary care visits, active ART prescriptions, and viral suppression [11]. However, the role of health literacy was not included in this analysis. Examining the health literacy component is important because patients with limited literacy may experience worse health outcomes. Furthermore, patients of varying health literacy levels may have different needs to be addressed by CHWs during visits, or require more time or number of visits with CHWs [20]. The purpose of the current study is to examine the effect of health literacy on clinical outcomes and utilization of CHW services among PWH enrolled in a CHW intervention. A secondary aim is to identify most frequently utilized CHW services within this PWH cohort to understand general needs within this patient population.

## Methods

### Data

The data for this analysis come from a HRSA-funded evaluation of a CHW program implemented in ten Ryan White HIV/AIDS-funded primary care locations in the United States [11, 12, 14, 19, 21]. The sites included various types of healthcare delivery organizations in eight states, including four academic medical centers, three Federally Qualified Health Centers (FQHCs), two AIDS service organizations (ASOs), and one city public health department. The HRSA evaluation component funded one full-time equivalent CHW at each site, although each site had different level of staffing, with one to three CHWs by leveraging other resources. A complete description of this evaluation has been previously published [11]. CHWs received 80 hours of broad CHW training and HIV specific-training, including 16 hours of HIV literacy to support patient education on HIV treatment and adherence strategies and understanding lab values such as CD4 counts and viral loads [21].

### Sample

This analysis includes data collected between 2016 and 2019 on 397 patients who were enrolled in the CHW program and program evaluation. Data were captured at baseline and 6-month follow-up visits, including demographic information, having at least one HIV primary care visit, ART prescription, and viral suppression. Clinical data on relevant comorbidities, such as mental health condition, substance use disorder, or hepatitis C diagnosis were also included. Follow-up data at the 6-month visit was collected from 209 PWH (53%), and patients without both baseline and follow-up data were excluded.

### Measures

For the current analysis, health literacy level was the primary independent variable and CHW utilization, purpose of CHW encounters, viral suppression, primary care visits, and ART prescription were the dependent variables. Health literacy level was measured with the Brief Health Literacy Screening Tool (BRIEF), a four question, validated measure with five response options: always, often, sometimes, occasionally, never [22]. The BRIEF tool categorizes health literacy into three groups: inadequate health literacy, marginal health literacy, and adequate health literacy [22]. The BRIEF ranges from 2 to 20, with 2–12 indicating inadequate health literacy, 13–16 marginal health literacy, and 17–20 adequate health literacy. The BRIEF was administered to CHW program participants enrolled in the evaluation at baseline and 6-month follow-up. For this analysis, baseline BRIEF score was used as the primary independent variable in predicting outcomes at 6 months post-intervention.

CHW encounters with a patient were recorded on an electronic encounter form. A maximum of one encounter per day could be recorded. CHWs also entered the purpose(s) of the encounter into the electronic database. For the analysis, the frequency of encounters over 6 months was grouped into three categories: low encounters (1–3 days), moderate encounters (4–9 days) and high/very high encounters (≥10 days). We categorized encounters based on distribution in the sample. The encounter form included the following purposes: coaching (e.g., help with HIV or non-HIV disease management or services, harm reduction education, HIV disclosure, safer sex, life skills), provision of emotional support, making appointment referrals, accompanying patients to appointments, assisting with concrete services (e.g., completing applications for benefits or obtaining cell phone), health care appointment reminders, transportation coordination, and updating care plans and medical records. Encounters were recorded daily after an CHW-patient interaction. For the analysis, these categories (purpose of CHW encounters) were condensed into the following: logistical support (making appointment referrals, health care appointment reminders, updating care plans and medical records), accompany to appointment, transportation coordination, concrete services, coaching, and emotional support.

Primary care visits were measured as having at least one HIV primary care visit at 6 months post-baseline (no visits recorded/at least one visit recorded). This dichotomous measure was determined through chart review. Similarly, having an active ART prescription at 6 months post-baseline (yes/no/unknown) was identified through chart review. Lastly, viral suppression was measured at 6 months post-baseline based on the lab’s lower limit for undetectable viral load (<200cpies/mL). This dichotomous measure was determined through chart review. Chart reviews were conducted by trained staff at the sites, including either research assistants familiar with HIV disease progression and clinical outcomes or the CHWs themselves.

### Statistical analysis

Descriptive analyses were conducted to characterize the study sample. Demographic composition of included and excluded patients was compared using paired t-tests (individual level) for continuous variables and chi-square tests for categorical variables to assess for threats to validity (Table A1). Main outcomes at 6 months – CHW utilization, purpose of CHW encounter, viral load suppression, HIV primary care visit, prescribed ART - were stratified by BRIEF health literacy category, using chi-square analysis to identify significant differences. Logistic regression models examined the association between BRIEF category and the following primary outcomes: purpose of CHW encounter, viral load suppression, HIV primary care visit, and prescribed ART. Linear regression models examined the association between BRIEF category and CHW utilization (frequency of visits), as well as purpose of CHW encounters and CHW utilization. Models were adjusted for patient demographic characteristics such as age, gender (male, female, other), race (Black, White, other), mental health diagnosis (yes/no), substance use disorder (yes/no), and hepatitis C diagnosis (yes/no). Models for clinical outcomes (viral load suppression, HIV primary care visit, prescribed ART) included site-level fixed-effects to account for correlation among variables based on clinic location. Site-level fixed effects were not included for CHW utilization and purpose of CHW encounter logistic regressions, as we assumed CHW services are correlated with and driven by client needs.

## Results

### Population characteristics

The average age of the study cohort was 40.5 years, with 65.6% identifying as male. The majority of participants were Black (74.5%), and 8.7% identified as Latinx. Most participants spoke English as their primary language, were currently housed, and unemployed. Within this cohort, 34% had a mental health diagnosis, 31% had a diagnosed substance use disorder, and 12% had Hepatitis C. There were no significant differences in demographic characteristics or BRIEF score at baseline between included and patients lost to follow-up (Appendix Table A1). Complete demographic characteristics of the study cohort stratified by BRIEF category are reported in Table 1.

**Table 1:** Demographic characteristics of study cohort by BRIEF category (*n* = 209)

	Inadequate		Marginal		Adequate		Total	
	N	%	N	%	N	%	N	%
**Age** (mean, SD)	44.84 ± 11.14		38.23 ± 13.70		39.9 ± 12.80		40.45 ± 12.84	
**Gender**								
Male	24	63.2	31	70.5	82	64.6	137	65.6
Female	14	36.8	11	25	43	33.9	68	32.5
Other	0	0	2	4.5	2	1.6	4	1.9
**Race**								
Black	28	73.7	31	70.5	96	76.2	155	74.5
White	8	21.1	9	20.5	27	21.4	44	21.2
Other	1	2.6	2	4.5	3	2.4	6	2.9
No Response	1	2.6	2	4.5	0	0	3	1.4
**Hispanic or Latinx**	5	13.2	6	14	7	5.6	18	8.7
**Language**								
Non-English	4	10.8	1	2.3	2	1.6	7	3.4
English	33	89.2	43	97.7	125	98.4	201	96.6
**Housing**								
Currently Housed	35	94.6	36	85.7	108	86.4	179	87.7
Not Currently Housed	2	5.4	6	14.3	17	13.6	25	12.3
**Employment**								
Employed	7	20.6	11	26.8	46	36.5	64	31.8
Unemployed	27	79.4	30	73.2	80	63.5	137	68.2
**Mental Health Dx**	16	42.1	17	38.6	38	29.9	71	34.0
**SUD**	12	31.6	13	29.5	41	32.3	66	31.6
**Hep C Diagnosis**	3	7.9	7	15.9	17	13.4	27	12.9

### Bivariate analysis of CHW and clinical outcomes at 6 months post-intervention

Complete results for bivariate analysis of CHW utilization, purpose of CHW encounter, clinical outcomes (viral load suppression, HIV primary care visit, ART prescription) by BRIEF category are reported in Table 2.

**Table 2:** CHW utilization, purpose of CHW encounter, and health outcomes by BRIEF category

	Inadequate		Marginal		Adequate		Total		***p***-value
	N	%	N	%	N	%	N	%	
**CHW Utilization**									
Low	16	42.1	11	25.6	50	40.7	77	37.8	0.186
Moderate	14	36.8	17	39.5	32	26.0	63	30.9	
High/Very High	8	21.1	15	34.9	41	33.3	64	31.4	
**Purpose of Encounter**									
Logistics	19	50.0	22	51.2	57	45.6	98	47.6	0.776
Accompany	14	36.8	8	18.6	39	31.2	61	29.6	0.165
Transportation	10	26.3	15	34.1	44	34.6	69	33.0	0.623
Concrete Services	22	57.9	25	56.8	65	51.2	112	53.6	0.683
Coaching	33	86.8	37	84.1	86	67.7	156	74.6	**0.016**
Emotional	37	97.4	37	84.1	110	86.6	184	88.0	0.133
**6-Month Clinical Outcomes**									
Viral Load Suppression	18	54.5	26	65.0	54	49.1	98	53.6	0.223
HIV Primary Care	32	84.2	43	97.7	109	85.8	184	88.0	0.080
ART Prescription	37	100.0	42	100.0	117	95.9	196	97.5	0.190

#### CHW utilization and purpose of encounter – BRIEF category

Just under half (42.1%) of individuals with inadequate health literacy at baseline were low CHW utilizers (1–3 visits), while 39.5% of participants with marginal health literacy were moderate utilizers and 40.7% of those with adequate health literacy were low utilizers.

Participants with adequate health literacy were significantly less likely to receive coaching from their CHW (67.7%) compared to those with inadequate (86.8%) and marginal (84.1%) health literacy (*p* = 0.016). There were no significant differences for purpose of CHW encounters between BRIEF categories for logistics, accompany to appointment, transportation coordination, concrete services, or emotional support.

#### CHW utilization and purpose of encounter – general

The average number of CHW encounters was 7.76 (SD 7.71) over the 6 month period. Approximately one-third of participants (37.8%) had low CHW utilization (1–3 encounters in 6 months), 30.9% had moderate CHW utilization (4–9 encounters) and 31.4% had high/very high CHW utilization (≥10 encounters). Emotional support (88.0%) and coaching (74.6%) were reported in the vast majority of CHW-patient encounters. According to the CHW encounter data, about half of patients received assistance with concrete services (53.6%; e.g., completing applications for benefits or obtaining cell phone) and logistics support (47.6%; e.g., making appointment referrals, health care appointment reminders, updating care plans and medical records). Transportation coordination (33.0%) and accompanying participants to appointments (29.6%) were the least common reported purposes of CHW encounters.

#### HIV clinical outcomes

There were no significant differences in viral load suppression, HIV primary care visit, and ART prescription between BRIEF categories. Notably, ART prescription at 6 months post-intervention was consistently high in all three literacy categories – with the lowest rate being 95.9% in the adequate literacy category (and 100% in both inadequate and marginal categories). As there was little variation in this outcome, the logistic regression was dropped from the multivariate analysis of HIV outcomes.

### Multivariable results

#### CHW utilization and purpose of encounter – general

When looking at total frequency of CHW encounters at 6-months, individuals receiving transportation coordination had 5.38 more CHW encounters than those not receiving transportation coordination (95% CI 2.96,7.79; *p* < 0.001). Individuals receiving help with concrete services had 3.28 more CHW encounters than those not receiving help with concrete services (95% CI 1.28, 5.27; *p* = 0.001). Individuals receiving coaching have 3.91 more CHW encounters than those who did not receive coaching (95% CI 2.13, 5.70; *p* < 0.001). Individuals receiving emotional support have 4.89 more CHW encounters than those not receiving emotional support (95% CI 1.96, 7.82; *p* = 0.001). Complete linear regression results for frequency of CHW encounters are listed in the Appendix (Table A2).

#### CHW utilization and purpose of encounter – BRIEF category

Participants with inadequate health literacy had 3.58 higher odds of receiving coaching from a CHW, relative to those with adequate health literacy (95% CI 1.14, 11.26, *p* = 0.029). BRIEF health literacy category was not a significant predictor of CHW utilization or other purposes of CHW encounters (logistics, accompany, transportation coordination, concrete services, emotional support). Complete results are reported in Table 3.

**Table 3:** Multivariable results for CHW utilization and purpose of encounter at 6 months

	CHW Utilization^**+**^	Logistics^^^	Accompany^^^	Transportation^^^	Concrete^^^	Coaching^^^	Emotional^^^
**BRIEF Category**							
Adequate	*ref*	*ref*	*ref*	*ref*	*ref*	*ref*	*ref*
Marginal	1.763 (1.642)	1.228	0.453	0.874	1.13	2.338	0.678
Inadequate	−0.518 (1.401)	1.154	1.186	0.595	1.329	3.580*	5.991
**Age**	0.005 (0.046)	1.003	1.02	1.008	0.997	0.98	0.997
**Gender**							
Male	*ref*	*ref*	*ref*	*ref*	*ref*	*ref*	*ref*
Female	−0.922 (1.289)	0.681	0.606	0.736	0.822	0.81	0.927
Other	0.876 (2.682)	0.826	0.765	2.47	1	1	1
**Race**							
Black	*ref*	*ref*	*ref*	*ref*	*ref*	*ref*	*ref*
White	−1.964 (1.669)	0.717	0.803	0.429	1.2	0.634	1
Other	−1.739 (1.557)	2.016	1.85	0.443	1.165	1.425	3.144
No Response	−3.533 (1.193)	0.23	1.223	0.838	0.599	0.939	1.795
**Mental Health Dx**	0.960 (1.599)	0.494*	1.183	2.296*	1.2	1.135	2.053
**SUD**	−1.806 (1.474)	1.081	0.983	1.213	0.709	1.425	0.529
**Hep C Dx**	2.088 (2.242)	0.655	2.951*	1.912	0.975	1.913	3.777

^+^Linear regression, standard errors in parentheses

^^^Logistic regression: exponentiated coefficients

* *p* < 0.05, ** *p* < 0.01, *** *p* < 0.001

*SUD* substance use disorder

#### HIV clinical outcomes

Individuals with marginal health literacy had 14.13 higher odds of an HIV primary care visit at 6 months (95% CI 1.07, 186.06; *p* = 0.044) relative to those with adequate health literacy. BRIEF category was not a significant predictor of viral load. Complete results are reported in Table 4.

**Table 4:** Logistic regression results for clinical outcomes at 6 months post-intervention

	Viral Load	HIV Primary Care Visit
**BRIEF Category**		
Adequate	*ref*	*ref*
Marginal	1.541	14.13*
Inadequate	0.835	1.293
**Age**	1.011	1.024
**Gender**		
Male	*ref*	*ref*
Female	1.451	0.197**
Other	0.915	0.0555
**Race**		
Black	*ref*	*ref*
White	1.459	14.32**
Other	1.758	1
No Response	1.372	1
**Mental Health Dx**	1.114	2.197
**SUD**	0.915	0.344
**Hep C Dx**	0.644	0.140**

Exponentiated coefficients; * *p* < 0.05, ** *p* < 0.01, *** *p* < 0.001

*SUD* substance use disorder

## Discussion

While the impact of health literacy on PWH health outcomes is frequently discussed, less is known about how health literacy level affects utilization of CHW services among this population. The results of the current analysis indicate health literacy level was significantly associated with receiving coaching from CHWs, specifically that PWH with inadequate health literacy had more coaching encounters with CHWs. Further, PWH with marginal health literacy were significantly more likely to have an HIV primary care visit at 6 months. PWH receiving transportation coordination, concrete services, coaching, and emotional support had a significantly higher frequency of CHW encounters than individuals who did not receive those services from CHWs. This suggests that these specific patient needs (transportation coordination, concrete services, coaching, emotional support) cut across health literacy levels and consistently require more assistance from CHWs in the HIV care setting.

Coaching, or “health coaching” is a commonly cited component of CHW work, in which CHWs provide counseling and assist with problem solving to promote self-management for the individuals they work with [23, 24]. In our study, CHW coaching consisted of help with HIV or non-HIV disease management or services, harm reduction education, HIV disclosure, safer sex, or life skills conversations. A recent systematic review identified 61 studies pertaining to types/characteristics of CHW interventions, 48 of which described CHWs in health education/coaching roles [24]. However, the majority of the CHW interventions focused on cancer prevention/treatment and cardiovascular disease [24]. In the context of diabetes management, CHWs have been particularly effective for individuals with low health literacy, who have significantly more visits with CHWs as compared to those with high health literacy [25]. Our study is the first to describe CHW coaching for PWH of varying health literacy levels. Our data show that in an HIV primary care patient population, those with inadequate health literacy had 3.58 higher odds of receiving coaching from a CHW, and had 3.91 more encounters with CHW, as compared to those who did not receive coaching. This suggests that individuals with inadequate health literacy receive more help navigating health care services and HIV-specific disease management, requiring more visits with CHWs than individuals with marginal or adequate health literacy. Our data provide support that CHWs play an important role in the HIV health care team in supporting patients, as we see no significant differences in rates of viral load suppression or HIV primary care visits at 6 months between health literacy categories in this cohort. Further research is needed in understanding the aspects of coaching that may directly or indirectly affect clinical outcomes.

Factors improving retention in care for PWH have been widely studied, and are an area in which CHWs have been effective [15, 26]. One study reported that among PWH who are racial/ethnic minorities with behavioral health comorbidities, those receiving peer support experienced significantly fewer gaps in HIV primary care [15]. Similarly, a systematic review of interventions with PWH as peers found positive effects with regard to linkage to care and retention in care [16]. Further, low health literacy can be a barrier for individuals with regard to retention in care [27]. However, a study assessing the effect of health literacy on HIV clinical outcomes found that individuals with poor health literacy did not have reduced levels of ART adherence or poor retention [28]. Similarly, our data show that individuals with lower health literacy did not have poor retention in care. In fact, individuals with marginal health literacy were significantly more likely to have an HIV primary care visit at 6 months post-intervention, relative to those with adequate health literacy. This suggests services provided by CHWs, such as coaching, may assist with retention in care for individuals with marginal health literacy. However, for PWH with lower health literacy, CHWs may be addressing other priority needs and medical care may be less of a priority.

It is known that PWH have complex and varied needs, both clinical and non-clinical, regardless of health literacy status [29, 30]. These needs, or social determinants of health (SDOH), encapsulate a wide range of economic, social, and environmental factors [31, 32]. In a study of 15,964 PWH in the United States, 23% reported at least one SDOH indicator, while 25% indicated 4 or more indicators [30]. Further, 31.7% had difficulties with transportation, which is consistent with the 32.5% of our cohort who received transportation coordination from a CHW [30]. Our data demonstrate that emotional support was the most frequently reported purpose of CHW encounter. Stigma associated with an HIV diagnosis can have a significant negative effect on mental health, as can unmet SDOH needs, which may explain why 88.2% of our cohort received emotional support from a CHW [33, 34]. Logistics support, such as making appointment referrals, health care appointment reminders, updating care plans and medical records), was also common in this cohort. PWH often require complex and multi-faceted care, as many have co-occurring mental and substance use disorders [35]. Similarly, nearly one third of the individuals in our cohort had a mental health diagnosis or substance use disorder. Consequently, logistical support, such as healthcare navigation and appointment reminders, is a much-needed service. In our cohort, CHWs provided logistical support to nearly half (46.9%) of all individuals, further underlying the importance and utility of this service for PWH. While patient navigators have filled this role in some settings, our study demonstrates that CHWs also assist PWH with these needs.

This study is not without limitation. First, while CHWs can help educate individuals and improve their HIV-related knowledge, this program was not designed to improve overall health literacy. No sites in this study screened for health literacy among CHWs, which could potentially strengthen programs in the future. Second, while the CHW program included training on educating individuals about treatment and how to read and understand lab results, our analysis did not directly measure changes in the client knowledge about HIV disease management or HIV health services. The focus of CHW intervention was to provide education, motivation, and support for treatment adherence which was included as part of coaching. Previous studies of CHW peer intervention have led to increase, but non-significant changes, in HIV knowledge during a 12-month period [15]. Future studies should include a knowledge score. Third, the individuals in this study were a convenience sample of those willing to work with a CHW and were enrolled in a non-randomized fashion. As there is no control group, the effect of health literacy on CHW utilization and health outcomes can only be compared among PWH who interacted with CHWs. However, there were no significant differences between individuals who stayed in the study versus those who were lost to follow up regarding demographic characteristics and health literacy. Fourth, this study follows individuals over a 6-month period, which is likely not long enough to truly ascertain the impact of health literacy on utilization of CHW services within this cohort. Lastly, this study includes individuals from ten different clinic locations. While we controlled for clinic location in our regression models, differences in populations and CHW program models across sites may affect the generalizability of these results. Despite the limitations, this paper makes important contributions to the literature. Our results indicated that CHWs can reach PWH populations across the health literacy spectrum.

## Conclusions

The integration of CHWs into HIV primary care clinics allowed for more detailed knowledge on the types of services rendered throughout this study and their effect on treatment outcomes. Health literacy level was significantly associated with receiving coaching from CHWs and having an HIV primary care visit at 6 months but did not affect viral load suppression or number of CHW encounters. The most common purposes of CHW encounters were emotional support, coaching, concrete services, and logistical support. The patient’s purpose of encounter was highly correlated with frequency of CHW encounters; however, health literacy status was not predictive of CHW utilization. This suggests individuals receiving these services may require more assistance from CHWs, regardless of their level of health literacy. Training CHWs to conduct comprehensive social needs assessment and screening for risk factors at the initial visit with clients can identify resources and guide CHW service delivery as part of the care team.

## Supplementary Information

**Additional file 1: Table A1.** Demographic Comparison of Included and Excluded Individuals

**Additional file 2: Table A2.** Linear Regression Results for Purpose of CHW Encounter

## Data Availability

The datasets generated and analyzed during the current study are not publicly available due the fact that they may compromise individual privacy. Any inquiries or requests for data can be made to the corresponding author.

## Abbreviations

CHW: community health worker
PWH: people with HIV
ART: antiretroviral therapy
HIV: human immunodeficiency virus
