# Supplementary material for: Health literacy, health outcomes and community health worker utilization: a cohort study in HIV primary care
Source: BMC Health Serv Res. 2022 Oct 17;22:1254. doi: 10.1186/s12913-022-08634-7 (PMC9578207; doi:10.1186/s12913-022-08634-7)
Supplement: Supplementary file 1 — Additional file 1: Table A1. Demographic Comparison of Included and Excluded Individuals [file 12913_2022_8634_MOESM1_ESM.docx]

Table A1: Demographic Comparison of Included and Excluded Patients

|  | **Included (N=209)** | | **Excluded (n=188)** | | **p-value** |
| --- | --- | --- | --- | --- | --- |
|  | **N** | **%** | **N** | **%** |  |
| **Age (mean, SD)** | 40.6±12.8 | | 42.2±11.9  16.5±4.3  18.2  20.4  61.3 | | 0.19 |
| **BRIEF Score (mean, SD)** | 16.3±4.2 | |  |  | 0.60 |
| Inadequate (%) | 18.2  21.1  60.7 | |  |  |  |
| Marginal (%) |  |  |  |  |  |
| Adequate (%) |  |  |  |  |  |
| **Gender** |  |  |  |  |  |
| Male | 137 | 65.6 | 133 | 72.7 | 0.08 |
| Female | 68 | 32.5 | 50 | 27.3 |  |
| Other | 4 | 1.9 | 0 | 0 |  |
| **Race** |  |  |  |  |  |
| Black | 155 | 74.5 | 152 | 83.1 | 0.13 |
| White | 44 | 21.2 | 23 | 12.6 |  |
| Other | 9 | 4.3 | 4 | 4.3 |  |
| **Latinx** |  |  |  |  |  |
| Yes | 18 | 8.7 | 12 | 6.52 | 0.45 |
| **Primary Language** |  |  |  |  |  |
| English | 201 | 96.6 | 174 | 94.6 | 0.29 |
| Non-English | 7 | 3.4 | 10 | 5.4 |  |
| **Housing Status** |  |  |  |  |  |
| Currently housed | 179 | 87.8 | 158 | 86.3 | 0.75 |
| Not currently housed | 25 | 12.3 | 25 | 13.7 |  |
| **Employment Status** |  |  |  |  |  |
| Employed | 64 | 31.4 | 54 | 32.9 |  |
| Unemployed | 140 | 68.6 | 110 | 67.1 |  |
| **Mental Health Condition** |  |  |  |  |  |
| Yes | 71 | 34.0 | 53 | 29.0 | 0.22 |
| **Substance Use Disorder** |  |  |  |  |  |
| Yes | 66 | 31.6 | 46 | 25.1 | 0.11 |
| **Hepatitis C Diagnosis** |  |  |  |  |  |
| Yes | 27 | 12.9 | 23 | 12.6 | 0.66 |
