# Supplementary material for: Health literacy, health outcomes and community health worker utilization: a cohort study in HIV primary care
Source: BMC Health Serv Res. 2022 Oct 17;22:1254. doi: 10.1186/s12913-022-08634-7 (PMC9578207; doi:10.1186/s12913-022-08634-7)
Supplement: Supplementary file 2 — Additional file 2: Table A2. Linear Regression Results for Purpose of CHW Encounter [file 12913_2022_8634_MOESM2_ESM.docx]

**Table A2: Linear Regression Results for Purpose of CHW Encounter**

|  | **Number of CHW Encounters** | | | | | |
| --- | --- | --- | --- | --- | --- | --- |
|  | **Logistics** | **Accompany** | **Transport** | **Concrete** | **Coaching** | **Emotional** |
| **Purpose of CHW Encounter** | 5.35 | 11.97 | 216.0*** | 26.54** | 50.13*** | 132.9** |
| **Age** | 0.956 | 0.961 | 0.971 | 0.972 | 0.982 | 0.97 |
| **Gender** |  |  |  |  |  |  |
| Male | *ref* | *ref* | *ref* | *ref* | *ref* | *ref* |
| Female | 0.596 | 0.836 | 1.032 | 0.569 | 0.627 | 0.66 |
| Other | 3.956 | 4.778 | 0.335 | 0.876 | 2.191 | 3.507 |
| **Race** |  |  |  |  |  |  |
| Black | *ref* | *ref* | *ref* | *ref* | *ref* | *ref* |
| White | 0.501 | 0.847 | 2.081 | 0.791 | 1.349 | 0.787 |
| Other | 0.0438* | 0.0598* | 0.154 | 0.119 | 0.0666* | 0.0447** |
| No Response | 0.19 | 0.266 | 0.285 | 0.417 | 0.393 | 0.237 |
| **Mental Health Dx** | 5.767 | 8.639 | 4.089 | 8.688 | 7.604 | 8.081 |
| **SUD** | 0.0973 | 0.187 | 0.258 | 0.294 | 0.154 | 0.293 |
| **Hep C Dx** | 3.725 | 1.998 | 2.202 | 2.925 | 1.715 | 1.444 |

Exponentiated coefficients; * p<0.05, ** p<0.01, *** p<0.001;
